# Schizophrenia

**DOI:** 10.1192/pb.bp.114.050328

**Published:** 2016-04

**Authors:** Jennie Elizabeth Higgs

As the author states in the introduction, there is no ‘typical person’ with schizophrenia. Each patient has differing needs and is burdened in various ways by symptoms or side-effects. The book aims to be relevant to all aspects of treatment, from managing first presentations to dealing with long-term complications of antipsychotic medication. It is important to note that it is not an introduction to the subject of schizophrenia. Knowledge of the definition and diagnosis of schizophrenia is assumed. This text is aimed at clinicians and is designed to be used as a tool in clinical practice by those who work with patients with schizophrenia.

The chapters take us logically from evaluation of acute psychosis to the stable phase via the issues of treatment resistance and comorbid substance misuse. The text is firmly evidence-based with reference lists in each chapter and any expert opinions clearly acknowledged. Clear subheadings contribute to the easy-to-read style and the guidance, especially in the chapter on evaluation and management of acute psychosis, is practical and safe. Using the subheadings as a framework for addressing a particular clinical situation would ensure that all aspects of mental and physical health as well as social and psychological issues would be covered.

Unfortunately, I do not feel it meets its aim of being ‘person centred’, and although it states that it is influenced by the recovery model, this aspect was not emphasised enough. No clear definition of recovery in schizophrenia is given and there is no guidance on how to go about supporting your patients in goal-setting. Neither is collaboration with the patient, which I associate with person-centred practice, emphasised.

The issue with this book is that it is aimed at the US market. It illustrates the differences between practice in the USA and practice in the UK, both in terms of pharmacological and non-pharmacological interventions.

